# Proteome dynamics and physiological responses to short-term salt stress in *Leymus chinensis* leaves

**DOI:** 10.1371/journal.pone.0183615

**Published:** 2017-08-28

**Authors:** Jikai Li, Guowen Cui, Guofu Hu, Mingjun Wang, Pan Zhang, Ligang Qin, Chen Shang, Hailing Zhang, Xiaocen Zhu, Mingnan Qu

**Affiliations:** 1 Animal Science and Technology, Northeast Agricultural University, Harbin, China; 2 Institute of Grass Research, Heilongjiang Academy of Agricultural Sciences, Harbin, China; 3 Institute of Plant Physiology and Ecology, Shanghai Institutes for Biological Sciences, Chinese academy of Sciences, Shanghai, China; Hainan University, CHINA

## Abstract

Salt stress is becoming an increasing threat to global agriculture. In this study, physiological and proteomics analysis were performed using a salt-tolerant grass species, *Leymus chinensis* (L. *chinensis*). The aim of this study is to understand the potential mechanism of salt tolerance in L. *chinensis* that used for crop molecular breeding. A series of short-term (<48 h) NaCl treatments (0 ~ 700 mM) were conducted. Physiological data indicated that the root and leaves growth were inhibited, chlorophyll contents decreased, while hydraulic conductivity, proline, sugar and sucrose were accumulated under salt stress. For proteomic analysis, we obtained 274 differentially expressed proteins in response to NaCl treatments. GO analysis revealed that 44 out of 274 proteins are involved in the biosynthesis of amino acids and carbon metabolism. Our findings suggested that L. *chinensis* copes with salt stress by stimulating the activities of POD, SOD and CAT enzymes, speeding up the reactions of later steps of citrate cycle, and synthesis of proline and sugar. In agreement with our physiological data, proteomic analysis also showed that salt stress depress the expression of photosystem relevant proteins, Calvin cycle, and chloroplast biosynthesis.

## Introduction

Soil salinity is an important abiotic stress that limits plant development, growth, productivity, and quality, especially in arid and semi-arid areas with high evapotranspiration rates [1, 2]. According to the soil map of the world by FAO/UNESCO, the area of 831 million hectares is affected by salt stress. Among them, area under sodic soils (alkaline soils) is 434 million hectares, while area under saline soils is 397 million hectares (http://www.fao.org/ag/agl/agll/spush/intro.htm). Soil alkalinity is usually associated with the presence of high exchangeable sodium in soil possessing high pH (≥8.5). Due to the hydrolysis of Na_2_CO_3_ and NaHCO_3_, plants grown on such soils not only suffer the toxicity of sodium, but also have high pH stress. The accumulation of salts in the soil causes a series of abiotic stress, such as osmotic stress, nutrimental imbalance, ion toxicity, and oxidative stress to plants [3].

To cope with such situations, plants have developed mechanisms to perceive the environmental stresses and respond through cellular, physiological and developmental changes to achieve an optimized growth and reproductive success. In fact, only a few plant species under such stressed conditions have successfully evolved a mechanism to keep them survived. For example, a xerophilous grass, *Leymus chinensis* (L. *chinensis*), is one of such species adapted to highly alkaline-sodic soil conditions. In general, L. *chinensis* is a perennial species of the Gramineae family. The strong rhizomes of L. *chinensis* facilitate to acclimate to saline-alkaline and dune conditions [4, 5]. Due to its high productivity and high protein content, this species is becoming major gramineous forage in Northern China and the Mongolian plateau, and it is a candidate grass extensively applied for the establishment or renewal of artificial grassland [6].

Salt stress regulates a ranges of gene expression in the cellular machinery, which leads to changes in the abundance of cellular proteins. Several studies have reported about large scale omics studies of L. *chinensis* such as genome-wide expression, EST and microarray under different abiotic stress [7, 8]. The availability of protein and gene databases along with the innovation of mass spectrometry have greatly promotes the developments of the proteomics approach, which is helpful to uncover the complex functions of model plants and crop species in response to various environmental stress [9].

Proteomic technologies are effective approach to conduct large-scale analyses on the proteomic profiles under stress conditions. For example, mass spectrometry (MS)-based proteomic analysis is a powerful tool for identifying the isoforms of specific proteins, and for distinguishing the specific and overlapping functions within a protein family. Extensive studies on physiological response, transcriptome, and proteomic analyses under salinity stress have focused on the long-term stress (≥48 h) [10–14]. However, very limited studies especially on proteome data focused on the early events (<24 h) in response to salt stress signals [13, 15]. Notably, previous studies have demonstrated that plants responded to salinity stress can be determined by the rapid perception of stress shock that occurs within a few hours [11, 13]. Thus, it is of importance to understand the specificity of functional proteins in response to stress shock of salinity in L. *chinensis* and understanding this is helpful for developing strategies to improve the tolerance of crops to complex multiple environmental stresses.

In the present study, NaCl were used as salt stress to imitate environment salt stresses. Seedlings of plants (L. *chinensis*) were experienced several gradient of NaCl concentration, and the concentration were ranged from 0 mM to 700 mM. The duration of NaCl treatments were 24 h and 48 h, respectively. We investigated the proteomic profiles of the early response of L. *chinensis* to salt stress using Label-free iTRAQ-tandem mass spectrometry (MS/MS) technology. We observed enrichment of membrane trafficking (V-ATPase), photosystem protein (PsbA), mitochondria respiratory pathways, i.e., citrate cycles, and osmoprotectants, such as proline, sugar and sucrose, suggesting that these proteins were crucial for escape-avoidance coping mechanism in the early response to the salt stress in L. *chinensis* leaves. Our results provided valuable insights into adaptive response of L. *chinensis* plants to salt stress treatments.

## Materials and methods

### Plant materials and NaCl treatments

Plump seeds of L. *chinensis* were sterilized by 5% sodium hypochlorite for 5 min, and washed 4 times in sterile distilled water for 12 h at room temperature, and then transferred to wet filter paper to germinate at room temperature (22–25°C) for 24 h. The uniformly germinated seeds were selected to grow in plastic pots containing Hoagland solution that was changed every two days. After 8 weeks (56 d after emergence), the plants were treated by NaCl with different concentrations, 0 mM, 100 mM, 200 mM, 300 mM, and 400 mM. 500 mM, 600 mM, and 700 mM for 24 and 48 h. After that, partially sampled leaves were used to measure the physiological parameters immediately and the remaining leaves were kept frozen in − 80°C for later use.

### Physiological parameters measurement

Soluble sugars and sucrose were measured as previously reported [16]. Proline concentrations were estimated using the ninhydrin reaction method [17]. Catalase (CAT) and peroxidase (POD) enzyme activity were measured by using the kits (Cat. nos. XG6, EY2, and FY3) supplied by Suzhou Keming science and technology co., Ltd. (China). Superoxide dismutase (SOD) enzyme activity was determined by the kit from the Nanjing Jiancheng Bioengineering Institute of Jiangsu Province, China (Cat. no. A001-3). Three biological replicates were used to minimize experimental error. Statistical significances of the differences were determined by Student's t-test by using R package (2.3.2) software.

### Hydraulic conductance measurements

The hydraulic conductance from roots to leaves (*C*_p_, 10^−8^ m^3^ s^−1^ MPa^−1^) was calculated as *U*_w_/(Ψ_s_ − Ψ_l_) as previously reported [18], where *U*_w_ (10^−8^ m^3^ s^−1^) is the water uptake rate of the whole plant, Ψ_s_ (MPa) is the water potential of the soil immediately outside the root, and Ψ_l_ (MPa) is the average water potential of the uppermost three leaves. Since plants were submerged the water potential of the soil solution, Ψ_s_ was regarded as 0. Three biological replicates were determined.

### Protein identification using HPLC-ESI-MS/MS

For proteomic analysis, the leaves in L. *chinesis* experienced to 0, 200, 400, and 600 mM NaCl treated for 24 h were sampled. The pretreatments process of samples was referred to [19]. Protein digestion using trypsin was performed according to the FASP procedure [20], and the resulting peptide mixture was labeled using the 4-plex / 8-plex iTRAQ reagent according to the manufacturer’s instructions (Applied Biosystems). Experiments were performed on a Q Exactive mass spectrometer coupled to Easy nLC (Proxeon Biosystems, now Thermo Fisher Scientific). MS/MS spectra were searched using MASCOT engine (Matrix Science, London, UK; version 2.2) against a nonredundant International Protein Index arabidopsis sequence database v3.85 (released at September 2011; 39679 sequences) from the European Bioinformatics Institute (http://www.ebi.ac.uk/). The search results by MASCOT for each SCX elution were further processed using the ProteomicsTools (version 3.05). The program Isobaric Labeling Multiple File Distiller and Identified Protein iTRAQ Statistic Builder were used to calculate the ratios of protein, in which Sample REF was used as reference, based on the weighted average of the intensity of report ions in each identified peptide. The final ratios of protein were then normalized by the median average protein ratio for unequal mix the different labeled samples as previously reported [20].

### KEGG and GO analysis

The sequence data of the selected differentially expressed proteins were in batches retrieved from UniProtKB database (Release 2014_02) in FASTA format. The retrieved sequences were locally searched against SwissProt database (plants) using the NCBI BLAST+ client software (ncbi-blast-2.2.28+-win32.exe) to find homologue sequences from which the functional annotation can be transferred to the studied sequences. In this work, the top 10 blast hits with E-value less than 1*e*-3 for each query sequence were retrieved and loaded into Blast2GO (Version 2.7.2) for GO mapping and annotation [21, 22]. Following annotation and annotation augmentation steps, the studied proteins were blasted against KEGG genes (Arabidopsis) to retrieve their KOs and were subsequently mapped to pathways in KEGG as previously reported [23].

## Results and analysis

In this study, we statistically analyzed interactions effects of NaCl concentration (0~700 mM) and salt treated duration (24 h and 48 h) on physiological traits, osmoprotectants, and ROS scavenging enzymes (Table 1). Majority of traits measured in this study were significantly affected by NaCl treatments, except for length of root and leaves across treated duration (24 h and 48 h). In terms of duration of treatments, ROS scavenging enzymes, i.e., POD, CAT and SOD, were significantly affected, while interactive effects of NaCl treatments and duration on SOD and POD were observed.

**Table 1:** Statistical analysis on interactive effects of duration and concentration of NaCl treatments on parameters identified relating to growth development and carbon metabolites.

Parameters	Time (24h/2d)	NaCl (mM)	Interaction
***Functional traits***			
**ROOT length (cm)**	0.02*	0.39	0.33
**LEAF length (cm)**	0.01*	0.68	0.26
***Physiological variables***			
**Hydraulic conductivity**	0.17	0.0005***	0.75
**Chlorophyll contents (mg m**^**-2**^**)**	0.08	7.64E-05***	0.72
***Carbon metabolites***			
**Proline contents (%)**	0.42	4.76E-05***	0.30
**Soluble sugar (%)**	0.24	0.0006***	0.19
**Soluble protein (mg g**^**-1**^**)**	0.08	7.64E-05***	0.72
***Redox reaction***			
**POD contents (U g**^**-1**^ **min**^**-1**^**)**	0.0003***	2.29E-05***	0.01**
**SOD contents (U g**^**-1**^**)**	1.17E-07***	2.00E-04***	0.002**
**CAT contents (U g**^**-1**^**)**	0.04*	0.71	0.10

*P* values, representing the significant levels of two factors (duration and concentration of NaCl) effects, were depicted as symbols “*”, “**”, and “***”, at P<0.05, 0.01 and 0.001, respectively.

**Fig 1:**
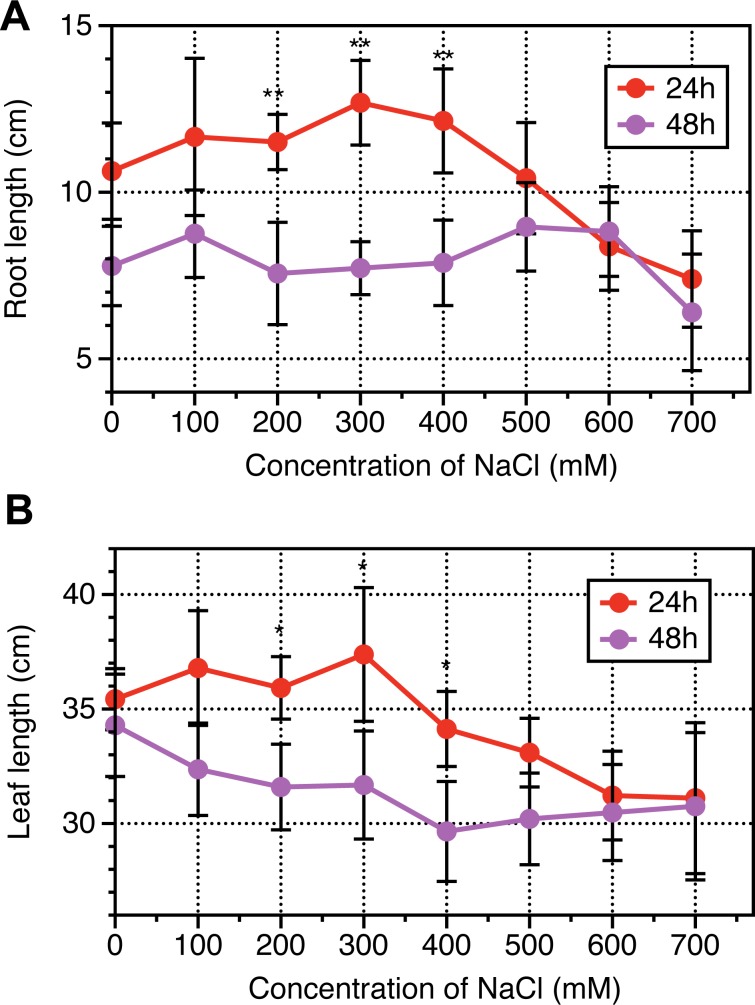
Dynamic response of root length and leaf length to a range of NaCl concentration for 24 hours and 48 hours in *Leymus chinensis*. Vertical bars represent mean values plus standard error values. Student *t*-test was used to compare significant differences between NaCl-treated duration of 24h and 48h, while symbol “*”, “**” represent P <0.05 and 0.01, respectively. At least 6 biological replicates were conducted.

**Fig 2:**
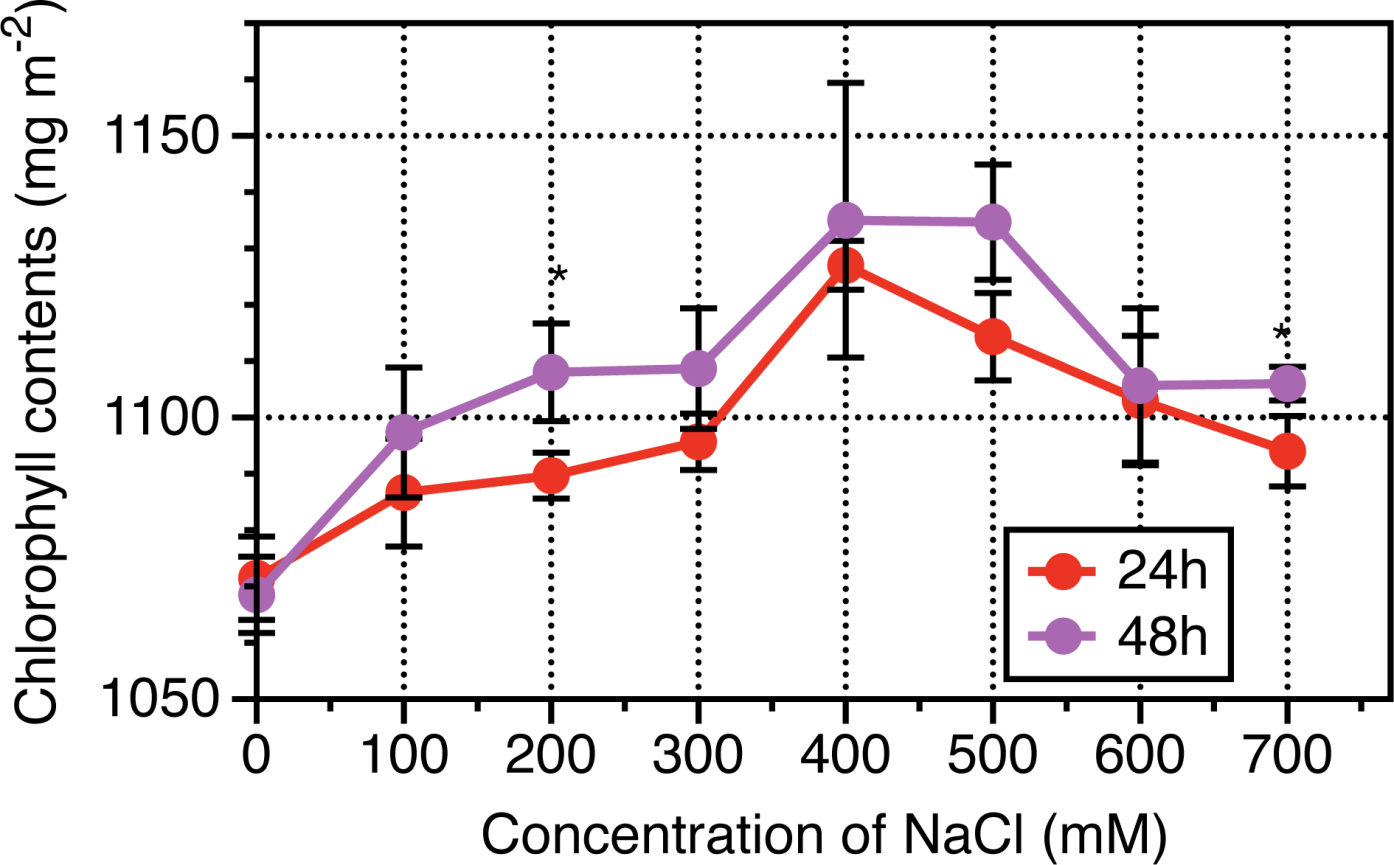
Dynamic response of leaf-based chlorophyll content to a range of NaCl concentration for 24 hours and 48 hours in *Leymus chinensis*. Vertical bars represent mean values plus standard error values. Student *t*-test was used to compare significant differences between NaCl-treated duration of 24h and 48h, while symbol “*”, “**” represent P <0.05 and 0.01, respectively. At least 5 biological replicates were conducted.

**Fig 3:**
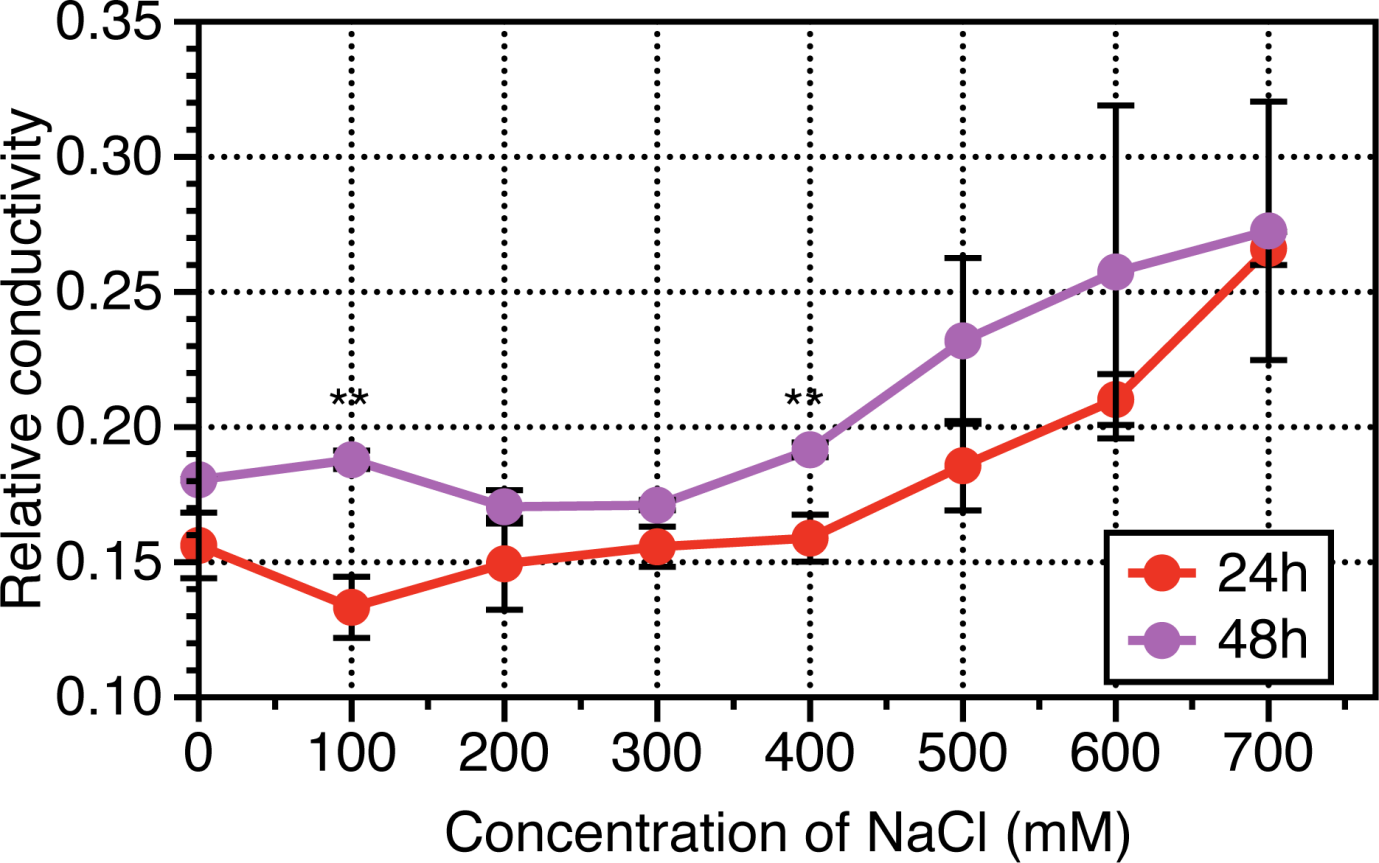
Dynamics response of relative hydraulic conductivity to a range of NaCl concentration for 24 hours and 48 hours in *Leymus chinensis*. Vertical bars represent mean values plus standard error values. Student *t*-test was used to compare significant differences between NaCl-treated duration of 24h and 48h, while symbol “*”, “**” represent P <0.05 and 0.01, respectively. At least 6 biological replicates were conducted.

The results of physiological dynamics revealed that root length and leave length increased following NaCl treatments from 0 to 400 mM NaCl, then sharply slumped when NaCl treated for 24 h, while in terms of NaCl treatments from 0 to 700 mM NaCl, no significant differences were observed when NaCl treated for 48 h (Fig 1). Leaf area based chlorophyll contents, representing photosynthetic capacity were investigated, and results revealed that Chlorophyll contents per leaf area were enhanced following with NaCl increased until 400 mM then decreased from 400 to 700 mM NaCl across NaCl durations (24 h and 48 h) (Fig 2). Interestingly, the chlorophyll contents of leaves experienced NaCl treatments for 48 h were higher than that for 24 h. The hydraulic conductance from roots to leaves were measured to determine NaCl stress induced osmotic adjustments at cellular levels. As indicated from Fig 3, the relative conductivity was increased with increase in the concentration of NaCl especially when its concentration was above 400 mM. The treated duration of NaCl at 48 h exhibited higher levels in relative conductivity than that at 24 h.

**Fig 4:**
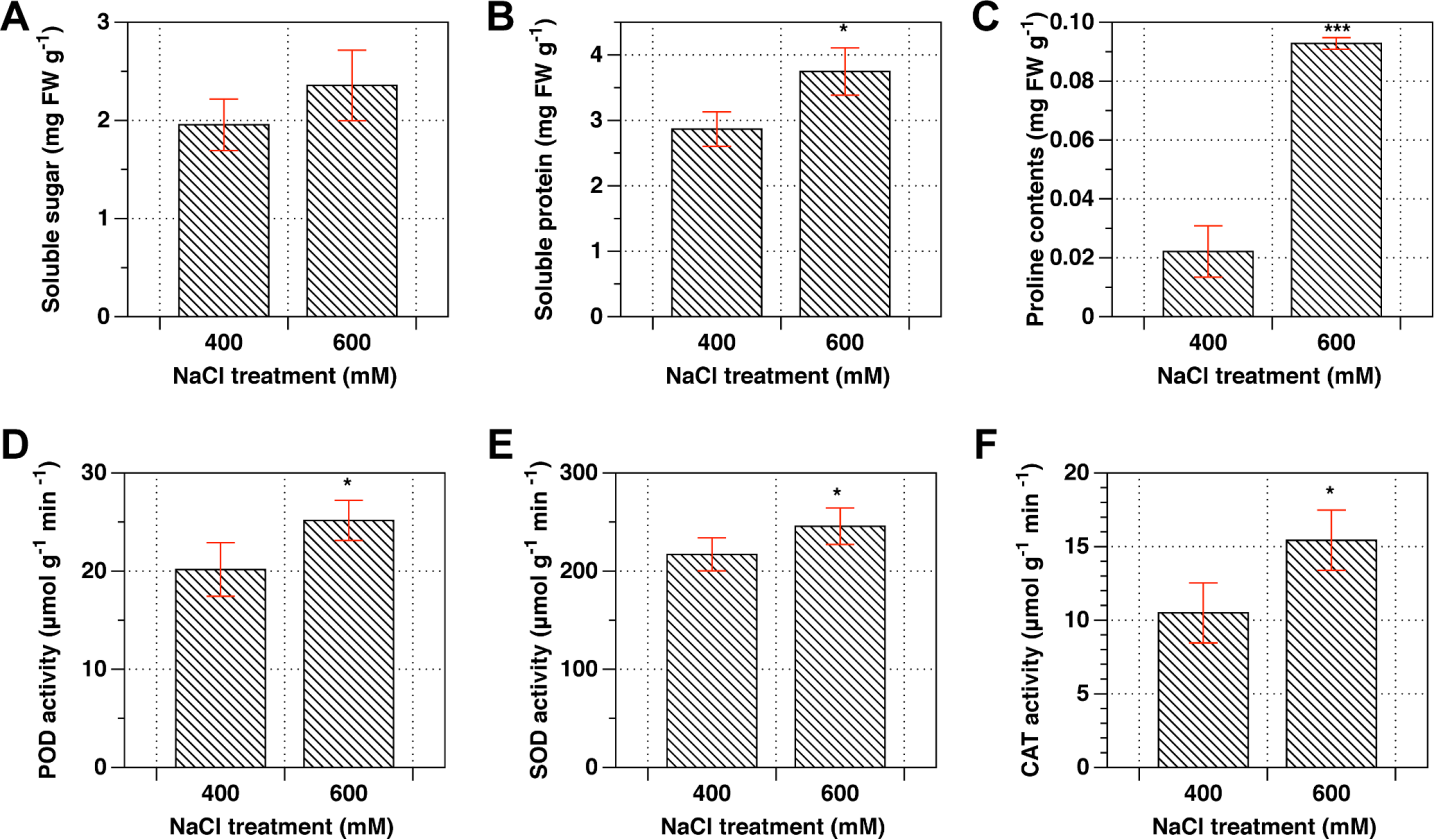
Comparison on the contents of osmoprotectants and activities of POD, SOD and CAT enzymes between 400mM and 600mM NaCl. Student *t*-test was used to compare significant differences between 400mM and 600mM NaCl concentration, while symbol “*”, “***” represent *P* <0.05 and 0.001, respectively.

Osmoprotectants, such as soluble sugars, sucrose, and proline contents are key abiotic stress indicators in plants. The comparison of response of these osmoprotectants, as revealed from Fig 4, was specifically represented between 400 mM and 600 mM NaCl concentrations. The contents of soluble sugar, protein, Proline were higher in 600 mM NaCl than that in 400 mM NaCl. The activities of POD, SOD and CAT were increased in 600 mM NaCl as well after NaCl treatments for 24 h.

**Fig 5:**
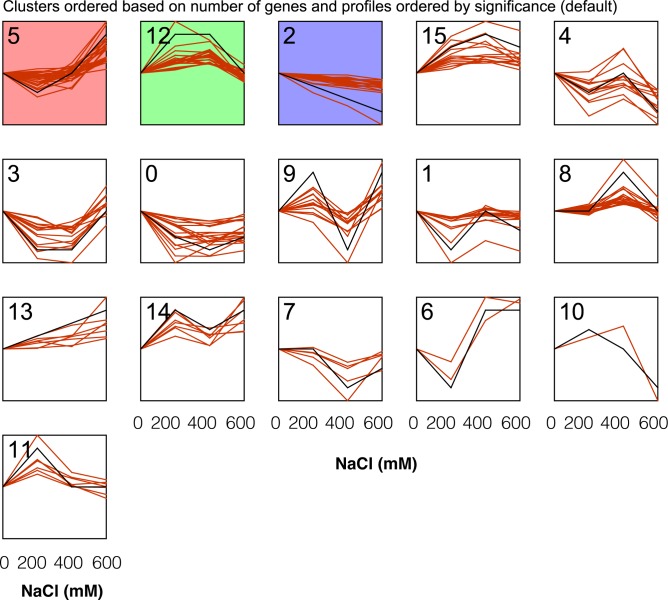
Classification of the patterns of dynamic response of 274 proteins to different NaCl concentrations. The colored panels represented the clusters enriched by significance, while black line in each panel represent the mean of expressed values of specific proteins in each group.

The proteomics were qualified by LTQ-MS, and 3814 proteins were identified with annotatation, and 274 proteins were differentially expressed across NaCl treatments (0, 200, 400 and 600 mM) based on one-way ANOVA analysis (see S1 Table). 15 groups were classified upon dynamics response of the 274 proteins to NaCl treatments. The ranking of groups was ordered by its significance (Fig 5). There were three common groups according to their significance, i.e., #5, #12 and #2. The three groups represent slow increase-fast increase, increase-plateau-decrease, and decrease continuously, respectively.

**Fig 6:**
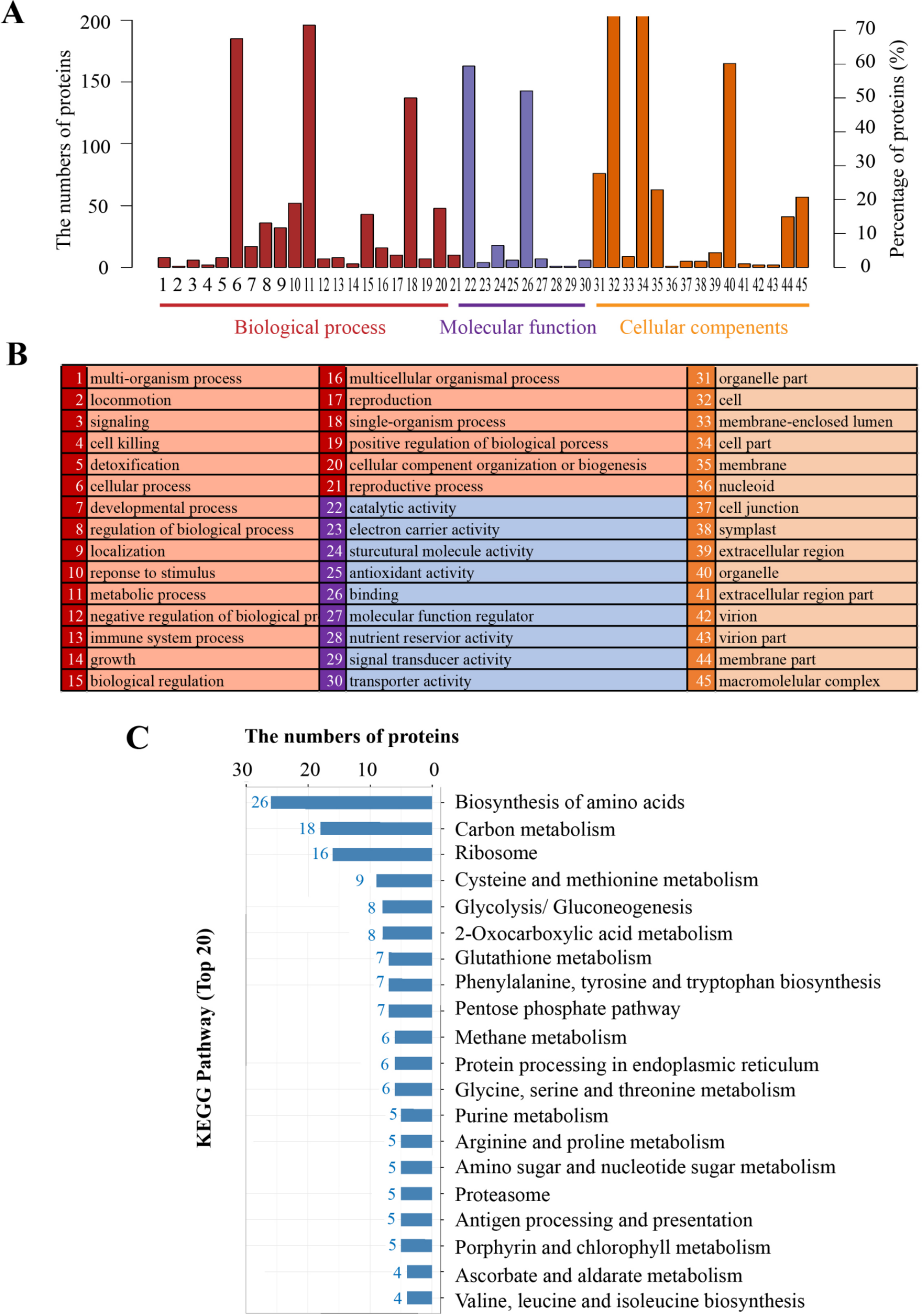
KEGG pathway and enrichments analysis on 274 annotated proteins with differential expression across a range of NaCl treatments. The numbers and percentage of proteins with significant enrichments (A) and the list of each biological category (B). Numbers of proteins corresponding to different metabolism pathways (C).

GO analysis on 274 proteins with significant differences across NaCl treatments (0, 200, 400 and 600 mM), and results showed (Fig 6A) that for biological process, the major enrichments were related to cellular process and metabolic process, while for molecular function, catalytic activity and binding were significantly enriched. In terms of cellular component, cell, cell part, and organelle levels were significantly enriched. Results of KEGG pathways indicated that the top two pathways containing 44 proteins in total, which are involved in the biosynthesis of amino acids and carbon metabolism (Fig 6B). Across the 44 proteins, there were 21 and 23 proteins showing down-regulation and up-regulations, in response to 600 mM over 400 mM NaCl treatments (Table 2).

**Table 2:** List of 44 proteins involving in carbon metabolism and biosynthesis of amino acids with differential expression in response to salt stress. Sensitivity represents the relative differences of proteins between 400mM and 600mM NaCl over 400mM NaCl, which is expressed as (A600-A400)/A400.

Seq. ID	Sequence descriptions	Score	MW [kDa]	calc. pI	Coverage	# Unique Peptides	400mM	600mM	Sensitivity
**C6YBD7**	Ribulose bisphosphate carboxylase oxygenase activase chloroplastic	856.24	40	6.93	53.33	1	1.59	0.73	-0.54
**W5I7D1**	ATP-dependent Clp protease proteolytic subunit-related chloroplastic	5.67	24.2	6.93	5.02	1	1.42	0.86	-0.39
**W4ZPS0**	ATP GTP binding	8.44	32.4	5.85	4.03	1	0.97	0.89	-0.09
**W5AFA2**	fructose-1,6- cytosolic-like	70.94	6.1	4.64	54.55	1	1.28	0.84	-0.35
**W5BLN6**	NADP-dependent glyceraldehyde-3-phosphate dehydrogenase	659	53	6.83	48.19	2	0.88	0.93	0.06
**W5FAL7**	Heat shock cognate 70 kDa 1	467.68	68.7	6.11	20.13	2	1.03	0.88	-0.14
**W5AK05**	Glucose-6-phosphate cytosolic	314.66	62.2	7.44	22.05	1	0.93	0.91	-0.02
**W5CDG5**	2,3-bisphosphoglycerate-independent phosphoglycerate mutase	490.66	56.2	5.68	39.61	3	0.99	0.93	-0.06
**W5DR81**	Calcium-transporting ATPase plasma membrane-type	71.46	113.5	5.05	1.81	1	0.8	0.86	0.08
**W5DSZ0**	ferredoxin-NADP(H) oxidoreductase	702.2	28.7	5.6	60.78	3	1.11	0.92	-0.17
**Q6XW17**	photosystem II 10 kDa polypeptide	260.19	10.2	8.65	52.53	4	1.1	0.98	-0.11
**W5F8D5**	Citrate synthase peroxisomal	249.68	22.7	9.32	40.4	6	1.05	1.03	-0.02
**W5EKJ6**	Fumarate hydratase chloroplastic	512.16	53.2	7.61	33.6	13	1.03	0.99	-0.04
**Q8RVZ9**	ferredoxin-NADP(H) oxidoreductase	600.38	38.8	8.1	44.48	3	1.09	1.07	-0.02
**W5C6B8**	2-oxoglutarate dehydrogenase E1 mitochondrial	130.03	91.5	7.05	8.08	2	1.05	0.96	-0.1
**W5I493**	NADH dehydrogenase subunit 9 (mitochondrion)	163.65	22.5	7.12	29.47	5	0.97	1.09	0.12
**Q1PBI3**	glucose-6-phosphate isomerase	383.05	62.2	7.46	24.87	2	0.84	1.15	0.36
**O24401**	Chlorophyll a-b binding chloroplastic	287.01	28.2	5.43	28.95	1	0.91	1.08	0.19
**W5EY15**	ATPase ASNA1	11.77	20.9	4.89	6.63	1	0.89	1.11	0.24
**W5FCR1**	NAD-dependent epimerase dehydratase	173.41	31.3	8.76	29.31	2	1	1.18	0.18
**W5F967**	Chlorophyll a-b binding chloroplastic	318.92	28	5.43	43.77	1	0.93	1.13	0.21
**W5BLL4**	Chlorophyll a-b binding chloroplastic	161.56	17.5	8.84	19.88	2	0.96	1.12	0.17
**W5A4D0**	sugar transporter	15.9	37.1	9.44	4.36	1	0.84	1.19	0.41
**W4ZSA0**	NADH dehydrogenase 1 alpha subcomplex subunit 1-like isoform X1	48.06	7.6	9.72	19.7	1	0.96	1.25	0.3
**W5I916**	photosystem I P700 apo A1	31.58	5.5	6.77	35.42	1	0.93	0.69	-0.28
**W5BNG7**	V-type proton ATPase subunit H	270.72	48.8	8.19	22.04	2	0.98	1.15	0.17
**W4ZSC8**	protochlorophyllide reductase B	385.61	42.2	9.17	30.38	5	0.91	0.88	-0.04
**W5HIA9**	transketolase 1	685.02	41.9	5.74	56.92	2	0.88	1.08	0.22
**Q8RYB1**	porphobilinogen deaminase (PBD)	229.05	33.2	5.74	27.27	7	0.91	0.75	-0.18
**W5FJ50**	Dihydrolipoyllysine-residue acetyltransferase component of pyruvate dehydrogenase complex	30.86	48.4	9.17	3.24	1	1.05	0.85	-0.19
**Q53UC8**	delta 1-pyrroline-5-carboxylate synthetase	112.06	77.5	6.55	8.38	4	0.94	1.16	0.23
**W5H9P8**	carbonic anhydrase	157.36	17.8	6.18	28.66	1	0.79	0.96	0.21
**T1WSS4**	glutathione peroxidase 4	16.94	21.3	7.03	5.88	1	0.77	1.02	0.32
**W5CQ46**	carbonic anhydrase	241.95	12.8	5.21	46.09	2	0.72	0.85	0.19
**W5FUB6**	Phosphoribosylformylglycinamidine cyclo- chloroplastic	133.47	34.3	4.93	18.63	5	1.05	0.98	-0.07
**W5ATW1**	S-adenosylhomocystein hydrolase 2	513.52	26	5.03	60.34	1	1.12	0.95	-0.15
**W4ZND2**	TPA: oxidoreductase	162.14	25.6	5.03	26.5	2	1.02	0.95	-0.07
**W5F5B0**	Bifunctional 3-dehydroquinate dehydratase shikimate chloroplastic	194.08	47.5	6.99	13.54	4	1.06	0.94	-0.11
**W5HVI8**	Phosphoserine chloroplastic	70.35	26.4	4.7	13.52	3	0.87	0.84	-0.03
**W5A1W2**	proline iminopeptidase-like	35.25	22.1	6.43	12.5	1	1	0.88	-0.12
**W5AEY8**	peptide methionine sulfoxide reductase chloroplastic-like	80.32	14.6	5.54	19.55	2	1.1	0.96	-0.13
**W5EL33**	Quinone oxidoreductase	89.67	40.5	8.6	7.85	3	1.15	0.93	-0.19
**W5F114**	Anthranilate chloroplast expressed	24.47	21.4	9.58	7.11	1	1.01	0.73	-0.28
**W5FMV4**	Lipoxygenase chloroplastic	68.18	49.8	5.86	5.09	1	0.88	0.63	-0.29

**Fig 7:**
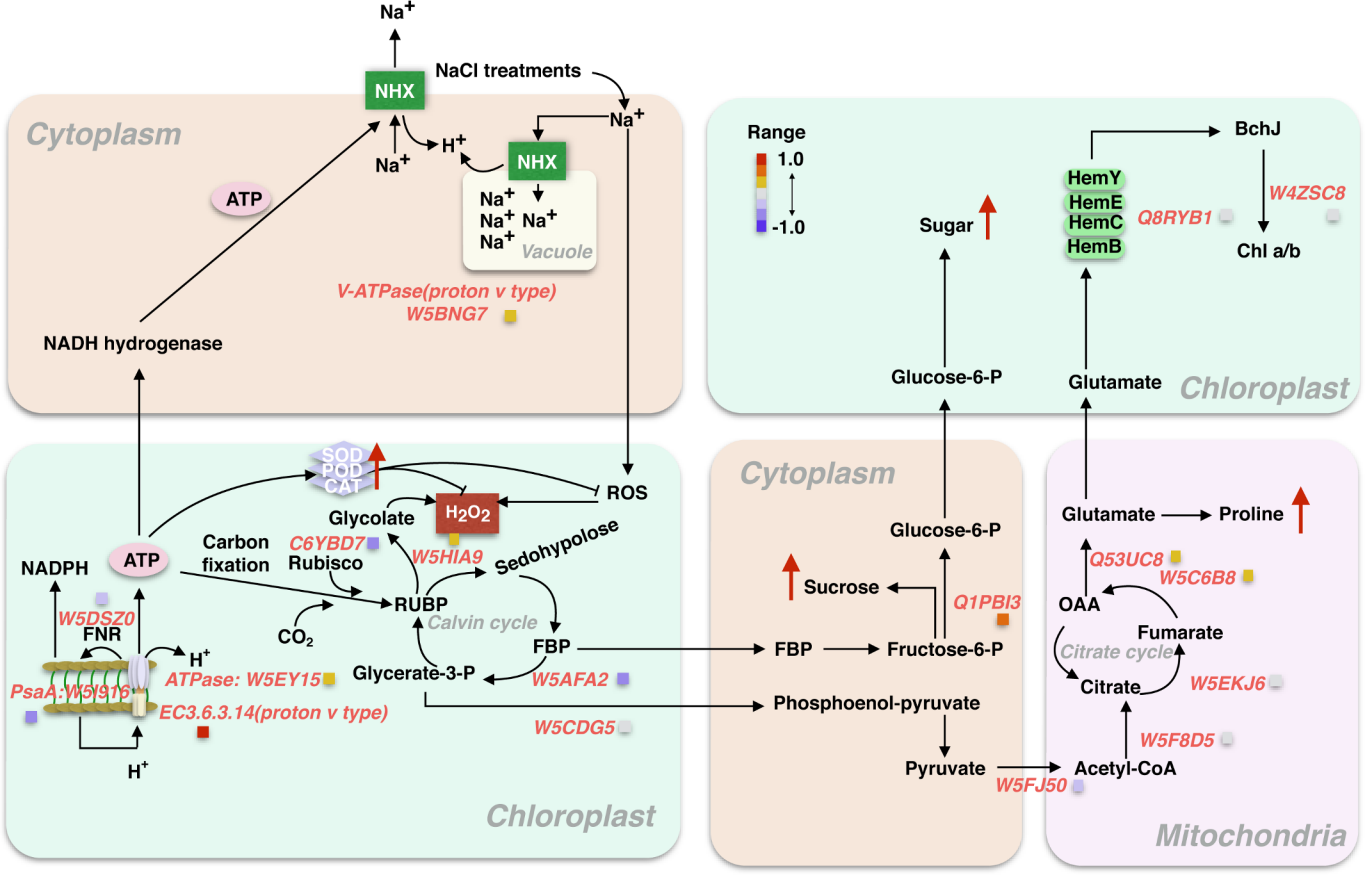
Schematic diagram summarized from the expression of 44 proteins relating to biosynthesis of amino acids and carbon metabolism in response to salt stress. For clear illustration, the evented organelle or regions at cellular levels were separated, while the color of light orange, light green and light pink represent cytoplasm, chloroplast and mitochondria, respectively. The red arrow indicated the relatively expressed levels of each component in 600mM NaCl compared with 400mM from independently experimental measurements.

The 44 proteins selected were used for further discussion. Taken together, as summarized from Fig 7, Na^+^ were absorbed from root to leaves. This evokes more ROS produced and H_2_O_2_ accumulated in cytoplasm which is probably detrimental for Rubisco kinetic properties and electron transfer chain. The overly accumulated H_2_O_2_ can be dissolved by SOD, POD and CAT, as indicated from Fig 7 that increase in activities of SOD, POD and CAT under 600 mM over 400 mM NaCl treatments. Biochemical reactions relating to CBB and photosystem proteins evented in Chloroplast were depressed by NaCl treatments, including Fructose-1,6 phosphate aldolase (W5AFA2) and PsaA (W5I916). In contrast, the expression of membrane localized proteins were increased due to the perception of cellular membrane and photorespiratory pathway were enhanced. These proteins include VATPase (W5BNG7), ATPase ASNA1(W5EY15) and transketolase (W5HIA9). As a result, sucrose in cytoplasm and starch accumulated in chloroplast were observed, this is directly or indirectly caused by increase in amounts of glucose-6-phosphate isomerase (Q1PB13). However, in mitochondria, there is not obviously alteration regarding several key protein amounts, such as DLAT—Dihydrolipoyllysine-residue acetyltransferase (W5FJ50), CSY- citrate synthase (W5F8D5), FUM- fumarate hydratase (W5EKJ6). Slight increase in conversation step from Fumarate to OAA including OGDH—2-oxoglutarate dehydrogenase (W5C6B8), Delta-1 -pyrroline-5- carboxylate synthase (Q53UC8) might be a reason explaining increased amounts of proline as shown from Fig 7.

## Discussion

L. *chinensis* is a promising model material for salt tolerance study [4, 5]. However, the mechanism underlying this salt-response process remains unclear. In this study, the dynamics of physiology and proteomics in response to salt stress in the leaves of L. *chinensis* seedling were analyzed. The 600 mM NaCl treated for 24 hours induced 16~46% reduction across roots and leaves as well as chlorophyll synthesis when compared with 400 mM NaCl (Figs 1 and 2). Bioinformatics analysis on Proteomics suggested that the 274 proteins were differentially expressed across 0, 200, 400 and 600 mM NaCl treatments, and of which, the biosynthesis of amino acids and carbon metabolism were significantly enriched, including 44 proteins. The shorten list were finally remapped to a framework of different biological pathways involving in Calvin cycle, citrate cycle, mitochondria respiratory pathways, accumulation of sucrose and synthesis of chlorophyll. We will briefly discuss our understanding on each pathway in response to salinity stress in L. *chinensis* seedling as followings.

### Chlorophyll biosynthesis and photosynthesis under salt stress

Chlorophyll biosynthesis in higher plants was carried and accomplished by sequential reactions, δ-aminolevulinic acid (ALA), porphobilinogen (PBG), uroporphyrinogen III (Urogen III), coproporphyrinogen III (Coprogen III), protoporphyrin IX (Proto IX), Mg-protoporphyrin IX (Mg ^2+^ Proto IX) and protochlorophyllide (Pchlide) were the major synthetic precursors during these sequential reactions [24, 25]. Salt stress can indirectly impair photosynthesis by depressing chlorophyll biosynthesis [26]. In our study, salt stress resulted in a 3% reduction of the chlorophyll contents in 600 mM over 400 mM NaCl treatments (Fig 3), which is consistent with previous studies that chloroplast development can be inhibited by severe salt stress [27].

Previously study on rice seedlings suggested that downregulation of Chlorophyll biosynthesis by salt stress could be attributed to decreased activities of Chlorophyll biosynthetic pathway enzymes [28]. Porphobilinogen deaminase (PBG deaminase) (EC-4.3.1.8) is one of most important enzymes of the pathway for chlorophyll and heme synthesis, which has been regarded as the key indicator for mirroring chlorophyll contents [28]. In current study, the 8% reduction in PBG deaminase (Q8RYB1) caused by 600 mM over 400 mM salt stress indicated that chloroplast biosynthesis pathway was inhibited to some extent (Table 2). This can lead to the accumulation of highly photosensitive photodynamic tetrapyrroles that produce singlet oxygen under stress conditions [28]. It is undeniable that the lack of chloroplast biosynthesis can cause the downregulation of photosynthesis, which is one of the primary processes affected by salinity [29, 30]. The decrease in photosynthetic capacity in salt-stressed plants might cause damage from the photosynthetic apparatus and photosystem proteins [30]

### Effects of salt stress on photosystem proteins

In this study, proteomic analysis showed that the expression of 4 proteins related to photosynthesis were significantly reduced by salinity stress especially in light reactions, including ferredoxin-NADP(H) oxidoreductase (FNR) (W5DSZ0) (decrease 17%), photosystem I P700 apo A1 (W5I916) (28% decrease) (PsaA), ATPase ASNA1(ATPaseASNA) (W5EY15) (24% increase), and V-type proton ATPase subunit H (ATPaseH) (W5BNG7) (17% increase) (Table 2; Fig 7). The accumulation of ATPaseASNA and ATPaseH under salt stress supported previous observations [31], suggesting that cyclic electron transfer (CET) reaction through FNR modulation were inhibited, which can affect dynamic response of transit OJIP chlorophyll fluorescence curve as reported previously [32].

### Effects of salt stress on RUBP carboxylation and regeneration in Calvin cycle

RCA is a key regulator of Rubisco activation in Calvin cycle. It is susceptible to salt stress, which may be due to reduction in carbon uptake caused by salinity stress as many previous reports [33, 34]. It was well known that Rubisco is the enzyme involved in the first step of CO_2_ uptake in Calvin cycle, the decrease of Rubisco activities could directly lead to a decrease in CO_2_ fixation efficiency as well as growth rates as shown in *A*. *lagopoides* [35], *Puccinellia tenuiflora* [36], *Kandelia*. *candel* [37]and *Tangut nitraria* [38]. In the current article, the contents of RCA after salt stress treatments 24 hours were decreased 54%, indicating the activities of Rubisco might be depressed during the salt stress process (Table 2). Another key enzyme associated with Calvin cycle, fructose-1, 6-bisphosphatase (W5AFA2), which catalyzes hydrolysis of fructose-1,6-bisphosphate to fructose-6-phosphate, was found downregulated 35% in response to salt stress (Table 2). Taken together, this explains why morphological traits such as leaves expansion rates and root growth rates were dramatically inhibited especially after 24 hours salt treatments (Fig 1).

### Effects of salt stress on citrate cycle

Plant respiration response to salt stress has been studied [39]. In mitochondrial respiratory pathway, Citrate cycle is one of most important metabolic pathway, which provides the major energy resources, such as ATP and NADPH for growth developments. In the initial step, pyruvate is converted to Ace-CoA from mitochondria to chloroplast through an acetyltransferase (W5FJ50). Our findings suggested that this acetyltransferase was reduced by 19%, which leads to downstream process, such as Fumarate hydratase (W5EKJ6), Citrate synthase (W5F8D5), and 2-oxoglutarate dehydrogenase (W5C6B8) reduced by 4%, 2%, and 10%, respectively (Table 2). Interestingly, our proteomic analyses showed that salt stress enhance the contents of proteins in Citrate cycle, especially for delta 1-pyrroline-5-carboxylate synthetase (Q53UC8) and 2-oxoglutarate dehydrogenase E1 (W5C6B8). The increase in glutamate can be further converted to proline. The increase in proline at 600 mM NaCl compared to 400 mM NaCl was confirmed by our independent measurements (Fig 4). The explanation for the accumulation of OAA is that pyruvate can be directly converted to OAA but at cost of 1 molecular ATP. Taken together, our results suggested that the citrate cycle plays key role in response to salt stress by regulating the expression of specific proteins as observed previously in other species [40].

### Damage repair and defense response under salt stress

Salinity can lead to cell membrane dehydration [41]. In order to avoid such dehydration, plants can reduce loss of water from the leaves by closing stomata or attempting to uptake more water from the soil [42]. Therefore, in order to regulate their water status, plants tend to adjust the responsiveness of stomata under abrupt changing environments, and modify their root water absorption capacity. In order to unravel the effects of salinity on cellular integration, we determined the hydraulic conductance in response to different concentration of NaCl (0, 200, 400 and 600 mM), and result showed that the stable increase in relative hydraulic conductance with increase of NaCl concentration (Fig 3). This indicates that salt-tolerance species, L. *chinensis*, can absorb more water from root to dissolve the gradients concentration of NaCl, which results in mitigating the high salinity stress.

Oxidative stress caused by excessive ROS is a direct indicator reflecting cellular damage caused by salt stress [43]. In order to determine whether antioxidant enzymes can promote ROS scavenging and mitigate the oxidative damage under salt stress in L. *chinensis* leaves, we measured the activities of SOD, POD and CAT. In general, POD, SOD and CAT are plant-specific enzymes involved in lignin formation, the cross-linking of cell wall components, and the removal of H_2_O_2_ against abiotic stresses [44, 45]. Our results showed that the activities of POD, SOD, and CAT were increased due to salt stress (Fig 4D–4F), suggesting that removal of H_2_O_2_ is needed when plants experience salt stress in L. *chinesis* leaves. This might be associated with salt-adaption response in plants as indicated previously [46, 47]. Meanwhile, many studies have shown that salt stress induced ROS accumulation might be a mechanism to protect plants rather than cause damage, at least at the initial stage [45, 48, 49].

### Effects of salinity on Amino acids

Extensive studies have demonstrated that proline, sucrose, and total soluble sugars are compatible osmolytes for the maintenance of turgor and for the stability of cellular structures under adverse environmental conditions [50–54]. In order to uncover the osmotic potential of cellular membrane, we have investigated contents of proline, sucrose and total soluble sugars. As shown in Fig 4A–4C, the proline levels, soluble sugar and soluble protein under 600 mM salt stress were 400%, 25%, and 36%, respectively, higher than 400 mM salt conditions. The accumulations of these osmoprotectants such as proline, sucrose and sugar under salt stress conditions were similar to that under drought conditions [33]. The V-ATPase (proton v type) (W5BNG7) were enhanced 17% under 600 mM NaCl compared with 400 mM NaCl (Table 2), suggesting that more sodium ions were accumulated in vacuole to maintain osmotic potential of cellular membrane. This evidence might be a suggrogant of salt adaptive response [50]. In agreement with this, we have confirmed that, as mentioned above, relative conductivity of cellular membrane increase gradually with increasing salt stress (Fig 3).

## Conclusion remarks

Our proteome data provides good evidence to support physiological data, such as the inhibition of leaf expansion and root developments, higher accumulation of sugar and sucrose. By analyzing 274 proteins with differential expression in response to salt stress, we summarized the proposed signal regulation networks in salt stress responses (see Fig 7 for the pathways). We speculated that V-ATPase under salt stress activate ion channels and pump Na^+^ into vacuole. Excessive accumulation of Na^+^ can be carried to the chloroplast, provoking ROS and H_2_O_2_ that inhibited Calvin cycle and initial steps of Citrate cycle. However, salt stress speeds up the later steps of mitochondria respiration in L. *chinensis* leaves, resulting in the accumulation of proline, which might be as a surrogate for adaptive response to salt stress.

## Supporting information

S1 Table Relative expression abundance of the representative 274 proteins with differential expression across NaCl treatments ranging from 0~600mM. (XLSX)
